# 3rd European Conference on Bioinorganic Chemistry, Eurobic 3

**DOI:** 10.1155/MBD.1995.243

**Published:** 1995


					FIRST ANNOUNC M NT
UROBIC 3
3rd European Conference on Bioinorganic Chemistry
August 4-11, 1996, Noordwijkerhout, The Netherlands
Organization- C.Veeger, W.R.Hagen, M.C.Feiters
(WageningenlNijmegen, The Netherlands)
15 plenary lectures, 10 thematic
sessions
microsymposia, poster
The organization stimulates the
scientists, it is intended that they
participation of young
will be partially refunded
Please request information at e-mail address
EUROBIC@ SECR.BC.WAU.NL
am interested in receiving the first circular for EUROBIC 3
Title, Name
Affiliation
Address
Zip code, Town Country
Telephone + Fax + E-mail
Please return this form to
Prof. C.Veeger, Department of Biochemistry, Wageningen
Agricultural University,
Dreyenlaan 3, NL-6703 HA Wageningen, The Netherlands
Telephone +.31.837082868, Fax +.31.837084801
E-mail Eurobic@secr.bc.wau.nl
(as from October Ist, 1995: Telephone +.31.317.482868, Fax
+.31.317.484801 )
243

				

